# Decoding Genetics of Congenital Heart Disease Using Patient-Derived Induced Pluripotent Stem Cells (iPSCs)

**DOI:** 10.3389/fcell.2021.630069

**Published:** 2021-01-21

**Authors:** Hui Lin, Kim L. McBride, Vidu Garg, Ming-Tao Zhao

**Affiliations:** ^1^Center for Cardiovascular Research, The Abigail Wexner Research Institute, Nationwide Children’s Hospital, Columbus, OH, United States; ^2^The Heart Center, Nationwide Children’s Hospital, Columbus, OH, United States; ^3^Division of Genetic and Genomic Medicine, Nationwide Children’s Hospital, Columbus, OH, United States; ^4^Department of Pediatrics, The Ohio State University College of Medicine, Columbus, OH, United States; ^5^Department of Molecular Genetics, The Ohio State University, Columbus, OH, United States

**Keywords:** congenital heart disease, human induced pluripotent stem cells, NOTCH signaling, hypoplastic left heart syndrome, genetic models of CHD

## Abstract

Congenital heart disease (CHD) is the most common cause of infant death associated with birth defects. Recent next-generation genome sequencing has uncovered novel genetic etiologies of CHD, from inherited and *de novo* variants to non-coding genetic variants. The next phase of understanding the genetic contributors of CHD will be the functional illustration and validation of this genome sequencing data in cellular and animal model systems. Human induced pluripotent stem cells (iPSCs) have opened up new horizons to investigate genetic mechanisms of CHD using clinically relevant and patient-specific cardiac cells such as cardiomyocytes, endothelial/endocardial cells, cardiac fibroblasts and vascular smooth muscle cells. Using cutting-edge CRISPR/Cas9 genome editing tools, a given genetic variant can be corrected in diseased iPSCs and introduced to healthy iPSCs to define the pathogenicity of the variant and molecular basis of CHD. In this review, we discuss the recent progress in genetics of CHD deciphered by large-scale genome sequencing and explore how genome-edited patient iPSCs are poised to decode the genetic etiologies of CHD by coupling with single-cell genomics and organoid technologies.

## Introduction

Congenital heart disease (CHD) is a leading cause of birth defect-related death and affects ∼1% of live births in the United States (Hoffman and Kaplan, 2002; Nees and Chung, 2019). CHD is characterized by morphological abnormalities in the cardiac chambers, septa and valves as well as the great vessels arising from the heart. Congenital malformations of all aspects of the heart have been described but the most common types of CHD can be classified into the following categories: (1) cardiac septation defects, (2) conotruncal and aortic arch artery anomalies, (3) right- and left-sided outflow tract obstructive defects, and (4) left-right abnormalities (heterotaxy) (Garg, 2006; Bruneau, 2008). Septation defects consist of atrial septal defects (ASD), ventricular septal defects (VSD) and atrioventricular septal defects (AVSD) while common conotruncal and aortic arch artery anomalies include tetralogy of Fallot (TOF), persistent truncus arteriosus and interrupted aortic arch. Right-sided outflow tract obstructive lesions include pulmonary stenosis and pulmonary valve atresia with intact ventricular septum (PA-IVS), whereas hypoplastic left heart syndrome (HLHS), aortic valve stenosis (AVS) and bicuspid aortic valve (BAV) are common left-sided outflow tract obstructive defects. Abnormalities in left-right signaling in the developing embryos affect cardiac looping, which is critical for proper alignment of the atria chambers to their appropriate-sided ventricles and great vessels. This disruption in proper signaling is associated with complex forms of CHD, such as double outlet right ventricle and double inlet left ventricle, clinically termed as heterotaxy syndrome (Kathiriya and Srivastava, 2000). Other major CHD that does not fit into the abovementioned categories includes isolated valve anomalies (e.g., Ebstein’s anomaly of the tricuspid valve and mitral valve prolapse), total anomalous pulmonary venous connection, anomalous coronary artery and patent ductus arteriosus.

Epidemiologic studies reveal that genetic factors are the predominant cause of CHD whereas environmental factors (exposures, maternal conditions, intrauterine environment, etc.) are also important contributors (Liu et al., 2013; Pierpont et al., 2018). In total, specific genetic and environmental factors can be identified in 20–30% of all CHD cases. Genetic mechanisms underlying the development of CHD are complex and remain elusive using current genetic approaches (Liu et al., 2017; Pierpont et al., 2018). There are limited animal models to study the developmental genetics of CHD, and transgenic mice carrying human variants do not always recapitulate the clinical phenotypes of CHD (Majumdar et al., 2019). Human iPSCs are derived from somatic cells (such as skin fibroblasts or peripheral blood mononuclear cells) and have the potential to generate all cell types in the body originated from the three germ layers (Takahashi et al., 2007; Yu et al., 2007). Compared to animal models, patient iPSCs are clinically relevant and also include the genetic background of the affected individuals in a disease-specific manner, thus providing a powerful tool for studying the contribution of a given genetic variant to CHD. Patient-specific iPSCs can be differentiated into cardiomyocytes, endothelial/endocardial cells, cardiac fibroblasts and smooth muscle cells, which makes it feasible to study complex genetic regulation and gene-environment interactions simultaneously in multiple cell types in the heart (Hu et al., 2016; Zhao et al., 2017a; Gifford et al., 2019). Recent studies demonstrate that genome-edited iPSCs are ideal platforms to elucidate the regulatory roles of non-coding genetic variants in the risk of coronary artery disease and to investigate the contribution of combinatorial interactions of multiple genetic variants to complex cardiovascular disease (Lo Sardo et al., 2018; Deacon et al., 2019).

In this review, we discuss the latest progress on genetic etiologies of CHD uncovered by the state-of-the-art technologies such as whole genome sequencing (WGS) and whole exome sequencing. We explore the fascinating perspectives on using patient-specific iPSCs and CRISPR genome editing to functionally study the genetic and epigenetic (environmental) determinants of CHD.

## Genetics of CHD

With the advance of massively parallel sequencing, genetics of CHD have been aggressively explored in the past decade. Large scientific efforts such as NIH-funded Pediatric Cardiac Genomics Consortium (PCGC) have been established to coordinate the investigation of genetic variants present in CHD patient population relevant to clinical outcomes (Pediatric Cardiac Genomics Consortium et al., 2013; Jin et al., 2017). The genetic basis of CHD can be grouped into two categories: syndromic CHD and non-syndromic (isolated) CHD (Pierpont et al., 2018). Syndromic CHD is defined as CHD with other congenital anomalies, neurodevelopmental defects and/or dysmorphic features. Syndromic CHD may be caused by aneuploidy, copy number variants (insertions or deletions > 1,000 nucleotides), or single gene defects. Down syndrome (trisomy 21) is a common chromosome anomaly, and 40–50% of these patients have various types of CHD, with cardiac septation defects being the most common. Turner syndrome is caused by complete or partial loss of an X-chromosome, and left-sided defects (coarctation of the aorta, COA), BAV and HLHS are present in 30% of these patients. 22q11.2 deletion syndrome is one of the most common copy number variants with deletion of more than 40 genes on chromosome 22. Outflow tract defects are present in 75–80% of 22q11.2 patients. Syndromic CHD caused by single-gene defects includes Alagille syndrome (variants in *JAG1* and *NOTCH2*) and Holt-Oram syndrome (variants in *TBX5*) (Basson et al., 1997; Li et al., 1997b; Table 1). Genetic contributors of isolated CHD have been emerging in the past two decades and most variants are located in genes that are involved in the molecular regulation of cardiac development. Syndromic and isolated CHD display distinct genetic architectures: *de novo* protein-truncating variants (PTVs) are significantly enriched in syndromic CHD whereas inherited PTVs are mostly derived from unaffected parents in isolated CHD (Sifrim et al., 2016; Jin et al., 2017).

**TABLE 1:** A summary of single-gene variants underlying CHD.

**Gene**	**CHD**	**Discovery methods**	**References**
*GATA4*	Atrial septal defect Atrioventricular septal defect Pulmonary stenosis Tetralogy of Fallot Ventricular septal defect	Linkage analysis PCR Targeted sequencing	Garg et al., 2003 Hirayama-Yamada et al., 2005 Okubo et al., 2004 Sarkozy et al., 2005 Tomita-Mitchell et al., 2007
*JAG1*	Pulmonary artery stenosis Tetralogy of Fallot	BAC FISH Linkage analysis PCR SSCP Targeted sequencing	Eldadah et al., 2001 Li et al., 1997a Mcdaniell et al., 2006 Oda et al., 1997
*MIB1*	Left ventricular non-compaction	PCR Targeted sequencing Transgenic mice Zebrafish reporter assays	Luxan et al., 2013
*NKX2-5*	Atrial septal defects Atrioventricular conduction block Ebstein’s anomaly Tetralogy of Fallot	FISH Linkage analysis PCR Targeted sequencing	Benson et al., 1999 Goldmuntz et al., 2001 Schott et al., 1998 Stallmeyer et al., 2010
*NOTCH1*	Aortic valve stenosis Bicuspid aortic valve Coarctation of the aorta Hypoplastic left heart syndrome Tetralogy of Fallot	*In vitro* expression assay Luciferase reporter assay Microarray Whole exome sequencing Whole genome sequencing	Durbin et al., 2017 Garg et al., 2005 Mcbride et al., 2008 Kerstjens-Frederikse et al., 2016 Stittrich et al., 2014 Zahavich et al., 2017
*PCDHA13* *SAP130*	Hypoplastic left heart syndrome	Mouse forward genetics Whole exome sequencing	Liu et al., 2017
*TBX5*	Atrial septal defect Ventricular septal defect	Enhancer reporter assay PCR Targeted sequencing Transgenic mice Zebrafish reporter assay	Basson et al., 1997 Li et al., 1997b; Mcdermott et al., 2005 Smemo et al., 2012

*BAC, bacterial artificial chromosome; FISH, fluorescence *in situ* hybridization; SSCP, single-strand conformation polymorphism.*

Pathogenic variants linked to isolated CHD primarily encode transcription factors, signaling molecules, structural proteins and epigenetic modifiers that are essential for normal cardiac development (Zaidi et al., 2013; Pierpont et al., 2018; Nees and Chung, 2019; Table 1). For instance, genetic variants in highly conserved transcription factors critical for cardiac development are found in both familial and sporadic cases of CHD. *NKX2-5* variants are present in patients with TOF and ASD with conduction delay (Schott et al., 1998; Benson et al., 1999; Goldmuntz et al., 2001; Stallmeyer et al., 2010). Pathogenic *GATA4* variants are associated with ASD, VSD, AVSD, pulmonary stenosis (PS), and TOF (Garg et al., 2003; Okubo et al., 2004; Hirayama-Yamada et al., 2005; Sarkozy et al., 2005; Tomita-Mitchell et al., 2007). A small subset of *GATA4* variant-induced cardiac malformations in humans are recapitulated in transgenic mouse models harboring the mutant human *GATA4* variants (Misra et al., 2012; Han et al., 2015).

Components of the NOTCH signaling pathway are linked to both syndromic and isolated CHD. *JAG1* variants are observed in ∼90% of Alagille syndrome patients whereas *NOTCH2* variants account for additional 1–2% of individuals with Alagille syndrome (Li et al., 1997a; Oda et al., 1997; Mcdaniell et al., 2006; Kamath et al., 2012). Loss-of-function variants in *JAG1* cause pulmonary artery stenosis and TOF with or without pulmonary atresia (Eldadah et al., 2001). Heterozygous mutations in *DLL4* (ligand) and *NOTCH1* (receptor) lead to Adams Oliver syndrome with CHD present in about 25% of these patients (Stittrich et al., 2014; Meester et al., 2015). Variants in *RBPJ* which interacts with the cleaved NOTCH1 protein to form a transcriptional complex, are also linked to Adams Oliver syndrome (Hassed et al., 2012). Of note, pathogenic *NOTCH1* mutations are linked to BAV, HLHS, AVS, COA, and TOF (Garg et al., 2005; Mcbride et al., 2008; Kerstjens-Frederikse et al., 2016; Durbin et al., 2017; Zahavich et al., 2017). Mechanistically, *NOTCH1* mutations reduce the ligand binding ability, interrupt the S1 cleavage of NOTCH receptor in the Golgi, and impair the epithelial-to-mesenchymal transition (Riley et al., 2011). In addition, germline mutations in *MIB1* which encodes an E3 ubiquitin ligase that promotes endocytosis of NOTCH ligands, lead to left ventricular non-compaction (LVNC) in autosomal-dominant pedigrees (Luxan et al., 2013). Myocardial *Mib1* mutations in mice cause the expansion of compact myocardium to proliferative immature trabeculae and interruption of chamber myocardium development.

The encyclopedia of DNA elements (ENCODE) project suggests that more than 80% of human genomic DNA has a biochemical function (Consortium, 2012). The majority of disease-causing variants identified by genome-wide association studies (GWAS) are located in non-coding DNA elements, many of which are embedded in the DNase I hypersensitive (open chromatin) regions (Maurano et al., 2012). GWAS in CHD have similar findings (Cordell et al., 2013; Hu et al., 2013; Mitchell et al., 2015; Hanchard et al., 2016). *De novo* variants in enhancer elements have been found in several human developmental defects including CHD and neurodevelopmental disorders (Short et al., 2018). For example, sequence variants in a limb-specific enhancer ZRS which is located nearly 1 Mb from its target gene *sonic hedgehog* (*Shh*) result in limb malformations such as preaxial polydactyly (Lettice et al., 2003). Copy number variants affecting topological associated domains have also been implicated in disrupting enhancers and causing developmental defects (Lupianez et al., 2015). Distal *cis*-regulatory elements have been identified in *TBX5*, of which variants are responsible for Holt-Oram syndrome (Mcdermott et al., 2005; Smemo et al., 2012). Among patients with Holt-Oram syndrome, three quarters have CHD, with ASD and VSD as the most common cardiac defects. A homozygous variant found in an enhancer about 90 kb downstream of *TBX5* is associated with isolated ASD and VSD in a cohort of non-syndromic CHD patients. This single-nucleotide variant compromises the enhancer activity driving expression of *TBX5* in the heart in both mouse and zebrafish transgenic models (Smemo et al., 2012). Recent WGS and chromatin immunoprecipitation sequencing have enabled researchers to expand the genetic variants in non-coding DNA elements that may have a regulatory role in controlling gene transcription during heart development (Zhao et al., 2017b; Richter et al., 2020). Non-coding *de novo* variants (DNVs) are significantly enriched in individuals with CHD and potentially exhibit transcriptional and post-transcriptional regulatory effects on genes critical for normal cardiac morphogenesis. Genetic architecture of CHD in cardiac regulatory non-coding DNVs will be further elucidated with the advance of WGS and precise genome editing technologies.

## Patient-Specific iPSCs for Modeling Genetics of CHD

Although a genetic etiology is identified in about 1/3 of CHD patients, experimental models to functionally validate genetic variants associated with CHD are far from perfect. Genetically engineered mice have been used for studying fundamental genetics of cardiac development for more than 25 years. Murine models are able to recapitulate some aspects of human cardiac development due to their similar stages of cardiac morphogenesis and adult cardiac structure (Majumdar et al., 2019). However, there are substantial differences in genomic content and physiology between humans and mice. Orthologous heterozygous variants sometimes do not reproduce similar CHD phenotypes when introduced into the mouse genome. Patient-derived iPSCs appear to provide a unique platform to study the genetic mechanisms of CHDs as they retain all the genetic information of the original affected individuals. Combined with CRISPR/Cas9 genome-editing, single-cell genomics, and cardiac organoid engineering technologies, patient-specific iPSCs would greatly complement the murine genetic models of CHD and illustrate novel perspectives on genetic etiologies of CHD for future precision diagnosis and treatment.

Human iPSCs are promising models for studying genetic mechanisms of isolated CHD caused by single-gene defects. In addition to cell-autonomous inherited cardiac disease such as long QT syndrome (Moretti et al., 2010; Itzhaki et al., 2011), ventricular tachycardia (Zhang et al., 2014; Sleiman et al., 2020) and dilated cardiomyopathy (Sun et al., 2012; Hinson et al., 2015), patient iPSCs have been employed to model several types of CHD, including BAV and calcific aortic valve disease (CAVD) (Theodoris et al., 2015), supravalvular aortic stenosis (SVAS) (Ge et al., 2012), cardiac septal defects (Ang et al., 2016), Barth syndrome (Wang et al., 2014), and HLHS (Hrstka et al., 2017; Yang et al., 2017; Miao et al., 2020; Table 2). Human iPSCs can be differentiated to the desired cardiovascular cell types relevant for studying different CHD (Protze et al., 2019), though the immaturity of iPSC-derived cardiomyocytes (iPSC-CMs) continues to be a challenge for recapitulating the physiological scenarios in the heart (Karbassi et al., 2020; Zhao et al., 2020b). Robust cardiac differentiation protocols have been optimized to generate subtype-specific (atrial, ventricular and nodal) cardiomyocytes for precision disease modeling (Zhang et al., 2011; Lee et al., 2017; Protze et al., 2017; Ren et al., 2019; Liang et al., 2020; Zhao et al., 2020a).

**TABLE 2:** Current iPSC models for studying disease mechanisms of CHD.

**CHD**	**Variants**	**Cell types**	**Disease phenotypes**	**References**
ASD VSD AVSD	*GATA4*	Cardiomyocytes	Impaired contractility Defects in calcium handling Abnormal mitochondrial functions	Ang et al., 2016
BTHS	*TAZ*	Cardiomyocytes	Irregular sarcomeres Abnormal myocardial contraction Excessive ROS generation	Wang et al., 2014
CAVD	*NOTCH1*	Endothelial cells	Defective epigenetic architecture Disrupted transcriptional response	Theodoris et al., 2015
HLHS	*NOTCH1* Unknown	Cardiomyocytes	Abnormal gene expression NO signaling deficiency Disorganized sarcomeres Reduced contraction force Decreased metabolic activity	Hrstka et al., 2017 Paige et al., 2020 Yang et al., 2017
HLHS	Unknown	Endothelial cells	Endocardial differentiation defects	Miao et al., 2020
LVNC	*MKL2 MYH7 NKX2-5*	Cardiomyocytes	*NKX2-5* is a genetic modifier Abnormal gene expression	Gifford et al., 2019
LVNC	*TBX20*	Cardiomyocytes	Defects in cardiac proliferation Abnormal TGF-β signaling	Kodo et al., 2016
PA-IVS	Unknown	Cardiomyocytes	Abnormal developmental trajectory Reduced contractility	Lam et al., 2020
SVAS	*ELN*	Smooth muscle cells	Less mature and contractile Higher proliferation ability in response to PDGF	Ge et al., 2012
VSD	*TBX5*	Cardiomyocytes	*TBX5* haploinsufficiency Disrupted gene regulatory network	Kathiriya et al., 2020

*The CHD subtypes, genetic variants, relevant cell types, and disease phenotypes are listed. ASD, atrial septal defect; AVSD, atrioventricular septal defect; BTHS, Barth syndrome; CAVD, calcific aortic valve disease; HLHS, hypoplastic left heart syndrome; LVNC, left ventricular non-compaction; PA-IVS, pulmonary atresia with intact ventricular septum; SVAS, supravalvular aortic stenosis; VSD, ventricular septal defect.*

Human iPSC models of CHD have employed major cardiac cell types such as cardiomyocytes (CMs), vascular smooth muscle cells (SMCs), and endothelial/endocardial cells (ECs) that can be derived from patient-specific iPSCs for laboratory research. These patient-derived cardiac cells carrying genetic variants enable researchers to study the disease mechanisms in a petri dish (Table 2). For example, pathogenic *GATA4* variants cause cardiac septal defects and cardiomyopathy. A heterozygous variant in *GATA4* (G296S missense) is linked to 100% penetrant ASD, VSD, AVSD or PS (Garg et al., 2003). Human iPSC-CMs from heterozygous *GATA4*-G296S patients display impaired contractility, defects in calcium handling ability and abnormal mitochondrial functions (Ang et al., 2016). Molecular analysis reveals that mutant GATA4 disrupts the recruitment of TBX5 which binds to cardiac super-enhancers and leads to dysregulation of genes related to cardiac septation. In another study, Theodoris et al. (2015) have derived iPSCs from patients with BAV and CAVD which are linked to *NOTCH1* haploinsufficiency. In iPSC-derived endothelial cells (iPSC-ECs), *NOTCH1* heterozygous nonsense variants disrupt the epigenetic architecture of NOTCH1-bound enhancers and cause the depression of anti-osteogenic and anti-inflammatory gene regulation networks in response to hemodynamic shear stress (Theodoris et al., 2015). Furthermore, the same group have recently utilized a combination of human iPSC technology, machine learning and network analysis to identify an efficacious therapeutic candidate XCT790 for preventing and treating aortic valve disease in a mouse model, demonstrating the prospective pharmacogenetic applications of CHD patient-specific iPSCs (Theodoris et al., 2020). Ge et al. (2012) have employed iPSC-derived smooth muscle cells (iPSC-SMCs) to investigate how elastin (*ELN*) gene variants lead to narrowing or blockage of the ascending aorta in SVAS. SVAS iPSC-SMCs harboring *ELN* variants are less mature and contractile, and show fewer networks of smooth muscle actin filament bundles compared to healthy controls. These SVAS iPSC-SMCs have a higher proliferation ability and migration rate in response to platelet-derived growth factor (PDGF), indicating that SVAS iPSC-SMCs recapitulate the pathological features of SVAS patients and may provide novel insights for future therapies.

**FIGURE 1:**
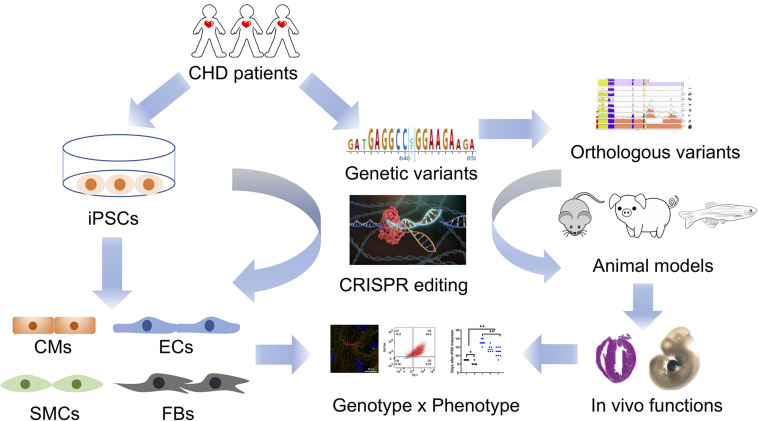
An integrated patient-specific iPSC model for studying genetics of CHD. Whole genome sequencing of CHD patients identifies prospective genetic variants that are retained in patient-specific iPSCs. Cardiovascular defects in CHD are recapitulated in patient iPSC-derived cardiac cells, such as cardiomyocytes (CMs), endocardial/endocardial cells (ECs), vascular smooth muscle cells (SMCs), and fibroblasts (FBs). Genetic variants associated with a CHD phenotype are corrected in diseased iPSCs and introduced to healthy iPSCs using CRISPR/Cas9 genome editing tools. These genome-edited iPSCs are further investigated to validate the cause-effect relationship between a given genetic variant and a CHD phenotype. In parallel, orthologous variants are genetically introduced into animal models (rodents, pigs, zebrafish, etc.) in order to investigate the *in vivo* effects of a given human variant on heart development. Together, an integrated model including both genome-edited human iPSCs and transgenic animals will yield a more comprehensive illustration of the genetic basis of CHD in the new era of genomic medicine.

Human iPSCs have been utilized to study complex genetics in CHD together with transgenic mouse models and clinical genetics. A recent study reveals that *NKX2-5* variants serve as a genetic modifier of a familial LVNC cardiomyopathy with variable age of presentation from childhood to incidental asymptomatic finding in adulthood (Gifford et al., 2019). Human iPSCs were created carrying the inherited compound heterozygous variants in *MKL2*, *MYH7* and *NKX2-5* while genetically engineered mice carrying the orthologous variants were also generated. By analyzing the phenotypes from transgenic murine hearts and patient iPSC-CMs, *NKX2-5* variants are identified as a genetic modifier for this cardiomyopathy with oligogenic inheritance. In another study, LVNC iPSC lines were generated from patients with *TBX20* variants (Kodo et al., 2016). LVNC iPSC-CMs show defects in proliferation which is caused by the abnormal activation of TGF-β signaling. In mice, overexpression of TGF-β1 leads to arrest in cardiac development, disturbed expansion of embryonic cardiomyocytes and trabecular/compact layer ratio in the left ventricle. Mostly recently, Kathiriya and colleagues have generated *TBX5* knockout human iPSC lines with different dosages (heterozygous and homozygous) and performed single-cell RNA sequencing and gene regulatory network analysis. *TBX5* haploinsufficiency alters the expression of CHD-related genes and reduced *TBX5* gene dosage disrupts gene regulatory networks in human iPSC-CMs. Abnormal genetic interaction between *Tbx5* and *Mef2c* leads to ventricular septation defects in transgenic mice with reduced *Tbx5* gene dosage (Kathiriya et al., 2020). These studies further highlight the combinatorial advantages of using human iPSCs and transgenic mouse models to reveal genetic mechanisms of CHD pathogenesis (Figure 1).

Hypoplastic left heart syndrome is a severe form of CHD characterized by aortic and mitral valve atresia or stenosis, leading to a hypoplastic left ventricle and aorta (Saraf et al., 2019). Though HLHS has a strong genetic component, the genetic etiology of HLHS is complex (Mcbride et al., 2005). Further, mouse models are not able to fully recapitulate the clinical phenotype (Liu et al., 2017; Grossfeld et al., 2019). Using HLHS patient-derived iPSC-CMs, multiple studies demonstrate the pathogenic link of *NOTCH1* variants to HLHS (Theis et al., 2015; Durbin et al., 2017; Hrstka et al., 2017; Yang et al., 2017). HLHS iPSCs harboring *NOTCH1* variants exhibit compromised ability to generate cardiac progenitors and HLHS iPSC-CMs show disorganized sarcomere structures and sarcoplasmic reticulum as well as a blunted drug response (Yang et al., 2017). Another independent study confirms that HLHS iPSCs have a deficiency in cardiomyocyte differentiation and NOTCH signaling pathway (Hrstka et al., 2017). Additionally, abnormalities in the nitric oxide (NO) pathway are found in the cardiac lineage specification of HLHS iPSCs with *NOTCH1* mutations. Small molecule supplementation could restore the cardiogenesis, implying a potential therapeutic target for HLHS patients. This study is consistent with the congenital cardiac abnormalities observed in *Notch1*^+/−^; *Nos3*^–/–^ transgenic mice and demonstrates that interaction between NO pathway and NOTCH signaling is required for proper development of the left-sided cardiac structures including the aortic valve (Bosse et al., 2013; Koenig et al., 2016). Recently, Miao et al. (2020) have highlighted the contribution of endocardial defects to the pathogenesis of HLHS using patient iPSCs and single-cell RNA sequencing of human fetuses with under developed left ventricles. Although the genetic causes of these HLHS iPSCs are unclear, endocardial defects lead to abnormal endothelial-to-mesenchymal transition, reduced cardiomyocyte proliferation and maturation, and disrupted fibronectin-integrin signaling. Another study by Mikryukov et al. (2021) has identified a critical role of BMP10 in the specification and maintenance of endocardial cells from human iPSCs. These iPSC-derived endocardial cells can induce trabeculation in iPSC-CMs and generate valvular interstitial-like cells, which are promising *in vitro* models for studying cardiac valve defects and LVNC. As the intercellular communication between endocardium and myocardium is essential for normal ventricular development (Macgrogan et al., 2018), further investigation would be warranted to illustrate how the abnormal crosstalk signaling leads to hypoplasia of the left ventricle using HLHS iPSC-CMs and iPSC-ECs.

The major challenge for studying genetics of CHD is lack of reliable models to functionally validate genetic variants that are discovered by massive genome sequencing. Although iPSC models are increasingly being used to study the contribution of genetic variation in the development of CHD, limitations should be carefully considered before any translational applications move forward. Human iPSC-CMs are fetal-like cardiomyocytes and show immature structural and physiological characteristics. For example, iPSC-CMs do not have mature structures of myofibrils and T-tubule, and they are misaligned compared to rod-shape adult cardiomyocytes (Karbassi et al., 2020; Zhao et al., 2020b). Enormous efforts have been made to promote the structural and functional maturation of iPSC-CMs, including the addition of thyroid and glucocorticoid hormones (Parikh et al., 2017), physical and electrical conditioning (Ronaldson-Bouchard et al., 2018), and co-culture with stromal cells in 3D cardiac microtissues (Giacomelli et al., 2020). In addition, iPSC-CMs are mostly cultured as a 2D structure which differs from the 3D structure of the human heart. Patient iPSC-derived cardiac organoids may be better models as a 3D substitute for the human heart (Rossi et al., 2018; Richards et al., 2020). However, it is still undetermined whether cardiac organoids can recapitulate the developmental scenarios of CHD pathogenesis. After all, any iPSC-based models are *in vitro* systems, which are fundamentally distinct from the *in vivo* environment. Although animal models best represent the *in vivo* environment, animals are different from humans in terms of physiology and genomics, and may not be clinically relevant. Therefore, we propose an integrated model which incorporates patient-specific iPSCs with transgenic animals (Figure 1). We envision that genetic variants associated with a CHD phenotype are tested in genome-edited iPSCs which are patient-derived and clinically relevant, while orthologous variants are also genetically introduced to animal models (rodents, pigs, zebrafish, etc.) to investigate the *in vivo* functions. The combination of human iPSCs and transgenic animals will provide us a more comprehensive illustration of pathogenetic mechanisms of CHD.

## Outlook

Recent advances in CRISPR/Cas9 genome editing (Adli, 2018), single-cell genomics (Tanay and Regev, 2017) and organoid (Rossi et al., 2018) technologies further propel the discovery of novel mechanisms of CHD development using patient- and disease-specific iPSCs. Precise genome editing technologies can be used to correct a given variant in patient iPSCs and then study whether the disease phenotypes can be rescued in genetically corrected isogenic cardiac cells (Hockemeyer and Jaenisch, 2016; Deacon et al., 2019). Concomitantly, this variant can be introduced to a healthy iPSC line with new genetic background to test whether it is sufficient to cause the disease phenotypes. Moreover, oligogenic inheritance in CHD may be studied in patient iPSCs by simultaneous correction or introduction of a combination of multiple genetic variants (Gifford et al., 2019). Single-cell RNA-seq analysis of human and mouse hearts has provided unprecedented resources on the trajectory of cardiac development *in vivo* at single-cell resolution and revealed a blueprint on how normal cell fate determination is altered under genetic perturbation and pathological conditions such as CHD (Cui et al., 2019; De Soysa et al., 2019; Litvinukova et al., 2020; Paik et al., 2020). Single-cell transcriptional profiling of healthy and diseased iPSCs during cardiac differentiation would decipher how a given genetic variant affects cardiac differentiation and developmental trajectories, and uncover new molecular insights in the pathogenesis of CHD (Churko et al., 2018; Kathiriya et al., 2020; Lam et al., 2020; Miao et al., 2020; Paige et al., 2020). As heart development is dependent on interaction among multiple cell types in the embryo, cardiac organoids and 3D bio-printing may serve as another tier of disease modeling using patient iPSCs (Lee et al., 2019; Nugraha et al., 2019). Cardiac organoids contain the spatial information of multiple cardiac cell types and lay out a 3-D platform to study the complex interactions between genotypes and phenotypes under normal and diseased conditions using patient-specific iPSCs. Although cardiac organoids have been used for modeling drug-induced toxicity and myocardial infarction (Richards et al., 2020), it is still challenging to generate cardiac structures such as heart valves and septa that can represent the developmental defects in CHD using the current cardiac organoid technologies. Future therapeutic breakthroughs in precision medicine of CHD would require the convergence of precision genome editing, single-cell genomics and cardiac bioengineering, which is built upon clinically relevant and patient-specific iPSC platforms.

## Author Contributions

HL and M-TZ: conception and design, figure preparation, manuscript writing, and final approval of manuscript. KM and VG: manuscript writing and final approval of manuscript. All authors contributed to the article and approved the submitted version.

## Conflict of Interest

The authors declare that the research was conducted in the absence of any commercial or financial relationships that could be construed as a potential conflict of interest.
